# The impact of manual rotation of the occiput posterior position on spontaneous vaginal delivery rate: study protocol for a randomized clinical trial (RMOS)

**DOI:** 10.1186/s13063-018-2497-7

**Published:** 2018-02-14

**Authors:** C. Verhaeghe, E. Parot-Schinkel, P. E. Bouet, S. Madzou, F. Biquard, P. Gillard, P. Descamps, G. Legendre

**Affiliations:** 1 Department of Obstetrics and Gynecology, Angers University Hospital, 49933 Angers Cedex, France; 2 Department of Biostatistics and Methodology, Angers University Hospital, 49933 Angers Cedex, France; 3 Mitovasc Institute, University of Angers, INSERM (French National Institute of Health and Medical Research) 1083, Angers, France; 4 CESP-INSERM, U1018, Team 7, Genre, Sexual and Reproductive Health, Université Paris Sud, 94807 Villejuif, France

**Keywords:** Manual rotation, Occiput posterior position, Posterior position, Anterior position, Vaginal delivery, Operative vaginal delivery, Cesarean, Cesarean section, Transabdominal ultrasound

## Abstract

**Background:**

The frequency of posterior presentations (occiput of the fetus towards the sacrum of the mother) in labor is approximately 20% and, of this, 5% remain posterior until the end of labor. These posterior presentations are associated with higher rates of cesarean section and instrumental delivery. Manual rotation of a posterior position in order to rotate the fetus to an anterior position has been proposed in order to reduce the rate of instrumental fetal delivery. No randomized study has compared the efficacy of this procedure to expectant management. We therefore propose a monocentric, interventional, randomized, prospective study to show the superiority of vaginal delivery rates using the manual rotation of the posterior position at full dilation over expectant management.

**Methods:**

Ultrasound imaging of the presentation will be performed at full dilation on all the singleton pregnancies for which a clinical suspicion of a posterior position was raised at more than 37 weeks’ gestation (WG). In the event of an ultrasound confirming a posterior position, the patient will be randomized into an experimental group (manual rotation) or a control group (expectative management with no rotation). For a power of 90% and the hypothesis that vaginal deliveries will increase by 20%, (10% of patients lost to follow-up) 238 patients will need to be included in the study. The primary endpoint will be the rate of spontaneous vaginal deliveries (expected rate without rotation: 60%). The secondary endpoints will be the rate of fetal extractions (cesarean or instrumental) and the maternal and fetal morbidity and mortality rates. The intent-to-treat study will be conducted over 24 months. Recruitment started in February 2017.

To achieve the primary objective, we will perform a test comparing the number of spontaneous vaginal deliveries in the two groups using Pearson’s chi-squared test (provided that the conditions for using this test are satisfactory in terms of numbers). In the event that this test cannot be performed, we will use Fisher’s exact test.

**Discussion:**

Given that the efficacy of manual rotation has not been proven with a high level of evidence, the practice of this technique is not systematically recommended by scholarly societies and is, therefore, rarely performed by obstetric gynecologists.

If our hypothesis regarding the superiority of manual rotation is confirmed, our study will help change delivery practices in cases of posterior fetal position. An increase in the rates of vaginal delivery will help decrease the short- and long-term rates of morbidity and mortality following cesarean section.

Manual rotation is a simple and effective method with a success rate of almost 90%. Several preliminary studies have shown that manual rotation is associated with reduced rates for fetal extraction and maternal complications: Shaffer has shown that the cesarean section rate is lower in patients for whom a manual rotation is performed successfully (2%) with a 9% rate of cesarean sections when manual rotation is performed versus 41% when it is not performed. Le Ray has shown that manual rotation significantly reduces vaginal delivery rates via fetal extraction (23.2% vs 38.7%, *p* < 0.01). However, manual rotation is not systematically performed due to the absence of proof of its efficacy in retrospective studies and quasi-experimental before/after studies.

**Trial registration:**

ClinicalTrials.gov, Identifier: NCT03009435. Registered on 30 December 2016

**Electronic supplementary material:**

The online version of this article (10.1186/s13063-018-2497-7) contains supplementary material, which is available to authorized users.

## Background

### Background and rationale

Posterior fetal presentations are encountered in the delivery room on a daily basis given that they make up 10–20% of fetal positions in the second stage of labor and 5–8% of fetal positions when the fetus is expelled [1–3].

Posterior positions are associated with a decreased rate of spontaneous vaginal deliveries. Indeed, the rate of instrumental delivery is estimated at 25–82% for posterior positions [1, 4–6]. Cesarean section rates are 44.4% for posterior positions, versus 4.2% for anterior positions [4, 5].

Spontaneous vaginal delivery is associated with lower morbidity rates, especially when the fetus is in an anterior position: there is a decrease in the rate of postpartum hemorrhage (PPH), infection, and perineal tear [4, 5]. Furthermore, vaginal delivery improves the patient’s satisfaction in the short and long term (higher satisfaction rates) [7, 8], with a decrease in psychological morbidity (lower rates of postpartum post-traumatic stress [9], baby blues, and postpartum major depression [10]). Spontaneous vaginal delivery improves the mother-child relationship, as has been evidenced by higher maternal breastfeeding rates when compared to cesarean section [11].

The management of labor should, therefore, focus on achieving spontaneous vaginal delivery in order to improve the mother’s wellbeing and health.

Manual rotation of a posterior position to an anterior position at full dilation is a common and accepted practice in obstetrics, especially as it seems that rotation using the Neville-Barnes forceps is no longer performed due to maternal and fetal complications, and is no longer taught [12, 13]. No current data recommends performing a rotation with other instruments (vacuum, spatula, etc.) in the event of a posterior position.

Manual rotation appears to be a simple method; Magnin therefore recommends trying to perform a systematic manual rotation for the posterior position. The efficacy of this maneuver was studied by Le Ray et al. in 2013 with a success rate of 90.1% and a vaginal delivery rate of 76.8% when performing manual rotation [14]. Although the results of this non-comparative study are encouraging, the external validity of manual rotation in this study is not assessable as an experienced team performed them.

Manual rotation seems to be associated with reduced rates of instrumental fetal delivery and maternal complications. In a retrospective study in 2006, Shaffer et al. demonstrated that cesarean section rate was lower among patients for whom a successful manual rotation was performed than among those who had a failed manual rotation with delivery in the occiput posterior (OP) position (2% vs 34%, *p* < 0.001) [15]. However, in Shaffer’s study, there is a lack of a control group without manual rotation, so it was, therefore, impossible to know whether this result was only due to manual rotation or to potential interfering factors. In 2010, Shaffer et al. compared manual rotation to expectant management in a retrospective study and found a significant decrease in the cesarean section rate (adjusted odds ratio (aOR) 0.12; 95% confidence interval (CI) 0.09–0.16 [16]). Nevertheless, there was a significant bias in this study because the “expectant management” group only consisted of patients with fetuses in the OP position during labor and not patients with fetuses delivering in the OP position at full dilation, a diagnosis of the type of posterior position was only made at birth. Patients presenting with a fetus with spontaneous rotation were therefore not taken into account. Finally, Le Ray et al. showed that manual rotation of a fetus in the posterior position in the second stage of labor (immediately at full dilation, at 1 or 2 h) significantly reduced the rate of instrumental vaginal delivery (23.2% vs 38.7% at 0, 1, and 2 h respectively, *p* < 0.01 [14]. However, no significant difference was found in terms of cesarean section rates. Many factors for bias, especially regarding selection, can be found in the study design, in comparing the practices between two different hospitals.

Therefore, it seems that manual rotation for posterior positions at full dilation is a simple and reproducible technique with presumably significant benefits in terms of spontaneous vaginal delivery rates. Some teams do not perform systematic rotations and prefer expectant management due to possible complications associated with attempting manual rotation, such as an abnormal fetal heart rate, cord prolapse, and emergency cesarean section [16–18].

### Benefits

Individual benefits for the experimental group (manual rotation) in comparison with the control group (expectant management) are:

- Increase in spontaneous vaginal delivery rates
- Lower rate of maternal and neonatal complications due to posterior presentation

The collective benefit is a reduced rate of instrumental vaginal deliveries and cesarean sections.

For the obstetricians, standardization of the technique is one of the primary expected benefits, along with Guidelines of Good Practice.

### Risks

The only potential risks related to manual rotation are:

- For the mother: the main risk is vaginal and cervical tearing [16, 17]. However, manual rotation results in less cases of postpartum hemorrhage and chorioamnionitis [16]. However, the duration of hospital stay is shorter [18] with manual rotation
- For the neonate: even if a pathological cardiotocogram (CTG) caused by manual rotation is found, Le Ray et al*.* found that only one emergency cesarean section was required in the case of a pathological CTG (out of 64 manual rotations) [17]. Shaffer et al. reported an Apgar score < 7 at 5 min in case of a successful manual rotation attempt when compared to no rotation [16]. The risk of umbilical cord prolapse exists if the maneuver is not performed correctly, in particular when the manual rotation is done with a thrust or if the head is repressed. This risk is low with only one umbilical cord prolapse per 368 manual rotations [19]

There is no reason for an unforeseen collective risk.

### Objectives

The primary objective of this study is to assess the impact of manual rotation on posterior positions at full dilation compared to expectative management (control strategy) in women whose term is ≥ 37 weeks’ gestation (WG).

Secondary objectives will be to assess the impact of manual rotation on posterior positions at full dilation compared to expectant management (control strategy) in women whose term is ≥ 37 WG, in terms of:

- Maternal morbidity and mortality
- Neonatal morbidity and mortality in the immediate postpartum period
- Duration of the second stage of labor, the delivery, and hospital stay

### Trial design

This was a monocentric, open, comparative, randomized study with two balanced, parallel groups (1 to 1). This superiority study will take place in a type-3 maternity ward in a French University Hospital in which 4000 deliveries are performed per year.

The manual rotation group will be compared to the corresponding control group for which an expectant management strategy will be used.

## Methods/Design

### Participants, interventions, and outcomes

#### Study setting

**Fig. 1:**
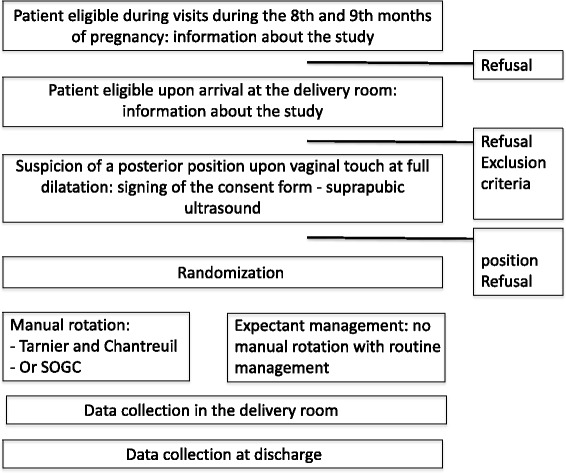
Participant timeline following Standard Protocol Items: Recommendations for Interventional Trials (SPIRIT) Figure format

The superiority study will take place in a type-3 maternity ward in a French University Hospital in which 4000 deliveries are performed per year. It will follow the SPIRIT guidelines (Additional file 1) which are presented in the Standard Protocol Items: Recommendations for Interventional Trials (SPIRIT) Figure in Fig. 1.

#### Eligibility criteria

#### Inclusion criteria

Pre-inclusion criteria upon arrival at the delivery room:

- Adult patient
- Singleton pregnancy
- Term greater than or equal to 37 WG
- Agrees to vaginal delivery upon entering the delivery room
- Cephalic presentation
- Clinical suspicion of posterior presentation at full dilation
- Epidural analgesia

Inclusion criteria at full dilation:

- Posterior presentation confirmed by ultrasound

Transabdominal ultrasound performed at full dilation by rotating the probe to the transverse plane above pubic symphysis. The midline angle and the fetal orbits will be used to determine the presentation. The presentations will be classified into two groups: posterior position if a fetal orbit is visible and anterior position if no fetal orbit is visible.

#### Exclusion criteria

Pre-exclusion criteria:

- Clinical suspicion of cephalopelvic disproportion
- Scarred uterus
- Presentation of the forehead or face
- Pathological fetal heart rate with a proven risk of acidosis required corrective surgery and second-line examinations
- Chorioamnionitis (suspected or proven)
- Temperature above 38 °C
- Pre-existing diabetes at pregnancy
- Fetal malformations or fetal coagulation disorders

Exclusion criteria at full dilation:

- Chorioamnionitis (suspected or proven)
- Hemorrhage during the first stage of labor
- Temperature above 38 °C during the first stage of labor

Exclusion of a patient is obligatory if, at any time, she withdraws her consent.

### Interventions

#### Experimental strategy

Manual rotation at full dilation in patients whose fetuses are delivered in the posterior presentation.

Two techniques are possible:

- Tarnier and Chantreuil’s technique: the right hand supports the back of the fetus’ right ear in the case of left posterior positions, and the left hand supports the back of the fetus’ left ear in the case of right posterior positions. The rotation movement is made towards the front, in the direction of the symphysis, during a thrust
- Technique of the Society of Obstetricians and Gynecologists of Canada (SOGC): the entire hand is placed in the patient’s vagina with the palm up; the fetal head is flexed and slightly dislodged. The occiput is rotated anteriorly by pronation or supination of the forearm

In both cases, manual rotation should be attempted during a contraction.

The choice of one technique over the other is made at the discretion of the senior obstetrician, given that neither technique has been shown to be superior [19].

#### Comparator

Expectant management, i.e., without manual rotation of the fetal head from a posterior to an anterior position. This approach is currently the standard practice in the department.

Operating physician: senior obstetrician.

During the entire inclusion period, the patient is not allowed to take part in another study involving a change to the approach. At the end of the study, regardless of whether it is prematurely closed or not, there is no exclusion period preventing the patient from taking part in another study.

### Outcomes

#### Primary endpoint

The primary endpoint will be the percentage of spontaneous vaginal deliveries in the two groups.

#### Secondary endpoints

Secondary endpoints will be the impact of manual rotation on posterior positions at full dilation compared to expectant management (control strategy) in women whose term is ≥ 37 WG, in terms of:

- Maternal morbidity and mortality
- Neonatal morbidity and mortality in the immediate postpartum period
- Duration of the second stage of labor, delivery, and hospital stay

#### Evaluation criteria

The only data collected at the time of pre-inclusion will be age, gravidity, and parity.

1. Evaluation criteria linked to the labor:
  - Time from the manual rotation to the birth (hours)
  - Duration of the second stage of labor (hours)
  - Term: weeks of gestation + days
2. Evaluation criteria associated with delivery:
  - Vaginal delivery/instrumental delivery/cesarean section
  - Blood loss: estimation using a collector bag (mean)
  - Performance of a right lateral episiotomy or lack thereof?: yes/no
3. Maternal data:
  - Perineal tears: third or fourth degree?: yes/no
  - Cervical lesions diagnosed by instrumental examination of the genital tract, requiring a suture
  - Clinically diagnosed vaginal thrombosis?: yes/no
  - Surgical lesions: ureter (wound, section), intestinal (wound), bladder (wound with insertion of urinary catheter)?: yes/no
4. Neonatal data:
  - Child’s sex: female/male
  - Child’s weight: grams
  - Apgar score
  - Arterial pH and lactate levels
  - Shoulder dystocia: yes/no/reduction through surgery

During the visit at discharge

1. Maternal data:
  - Blood transfusion?: yes (number of packed cells)/no
  - Postpartum endometritis: diagnosed based on the combination of fever, pelvic pain, and an infectious/biological disorder?: yes/no
  - Maternal fever ≥ 38.5 °C on two occasions (24 h following delivery)?: yes/no
  - Episiotomy wound healing disorder?: yes/no
  - Phlebitis/EP: diagnosed following Doppler ultrasound of the lower limbs or computed tomography (CT) angiography? yes/no
  - Occlusion?: yes/no
  - Fistula?: yes/no
  - Assessment of post-natal stress using a standardized questionnaire (evaluation of post-traumatic stress)
  - Transfer to the intensive care unit (ICU) department?: yes/no/reason
  - Duration of hospital stay: in days, following delivery
2. Neonatal data:
  - Intubation, ventilation > 24 h?: yes/no
  - Nasogastric tube feeding > 4 days?: yes/no
  - Intensive care > 4 days?: yes/no
  - Phototherapy in the case of hyperbilirubinemia: number of sessions
  - Clinically suspected fracture diagnosed using x-ray?: yes/no
  - Intraventricular or cerebral hemorrhage diagnosed using cranial ultrasound?: yes/no
  - Neonatal transfusion: number of packed cells
  - Ischemic encephalopathy diagnosed using magnetic resonance imaging (MRI) scan?: yes/no
  - Department and duration of hospital stay in days
  - Perinatal death (i.e., up to 7 days following delivery)

### Sample size

According to the hypothesis of Le Ray et al.’s study in 2013, 214 patients are required to show a 20% increase in the percentage of spontaneous vaginal delivery (60% without manual rotation vs 80% with manual rotation) with a power of 90% and an alpha risk of 5% (107 per group). We plan to include 238 patients in total to take into account the 10% of patients lost to follow-up.

### Recruitment

Eligible patients will initially be informed about the study during visits in the eighth and ninth months of pregnancy based on the various assessable selection criteria at the time. The information will be provided by a midwife or senior obstetrician (interview and delivery of the information letter written in a language that the patient understands). If the patient refuses, this will be noted in the patient’s medical records so that she is not asked to take part in the study at the time of delivery.

During the first stage of labor, following administration of the epidural, if the patient meets the study’s pre-selection criteria and if a posterior position is suspected by vaginal touch, the investigator will collect the patient’s consent following an interview. Information about the study will be reiterated in this interview. At least two copies of the informed consent form will be signed by all parties.

At full dilation, a suprapubic transabdominal ultrasound will be performed to confirm the posterior presentation.

If the ultrasound confirms the posterior position diagnosis and if the patient is still eligible (no exclusion criteria), the patient’s inclusion will be confirmed and the patient will be randomized.

In the case of randomized patients in the manual rotation group, the manual rotation will be performed immediately following randomization.

In the case of patients randomized in the expectant management group, no manual rotation will be performed and labor will proceed in line with standard practice.

## Assignment of interventions

### Allocation

Strategy allocation will be performed by randomization stratified by parity (primigravida/multigravida). The randomization procedure will be carried out by the Biostatistics and Methodology Department of Angers University Hospital and it will be generated digitally. From the time of their inclusion, patients will be randomized using a web-based system (Clinsight® software) and receive the corresponding care.

### Blinding

Only statistical analysis will be blinded.

## Data collection, management, and analysis

### Data collection methods and management

All information required by the protocol will be recorded in the case report forms. Data will be collected as it is obtained and will be recorded in the digital case report form. Comments will be added to justify or explain missing data or values that are outside the expected norms.

The digital case report form will be compiled using an Internet-based tool for data collection. Investigators will be provided with a document to help them use this tool. Each investigator will be responsible for the accuracy, quality, and relevance of all data recorded.

Any changes will be subject to an audit trail that will log all amendments made since the data was initially recorded. In the event of an amendment, the investigator may be asked to provide the reason for changing the data. At the end of the study, the investigator will be asked to provide a paper copy that they have authenticated (dated and signed). The investigator must archive a copy of the authenticated document to be given to the sponsor.

The following documents will be archived at the Promotion Unit of Angers University Hospital and in the buildings of the Department of Obstetrics and Gynecology of Angers University Hospital until the end of the period of practical use, in accordance with the regulations in force. These documents are:

- Protocol and appendices, unforeseen amendments
- Signed consent forms
- Individual data (authenticated copies of raw data)
- Follow-up documentation (monitoring)
- Records of serious adverse events (SAEs)

### Statistical analyses

Final report of the study or summary of the final report. At the end of the period of practical use, all the documents to be archived will be placed under the responsibility of the sponsor and the principal investigators for 15 years following the end of the study, in accordance with institutional practices. Nothing may be destroyed without the agreement of the sponsor. After 15 years, the sponsor will be consulted regarding the destruction of data. All data, documents and reports may be subject to audit or inspection.

### Statistical methods

**Fig. 2:**
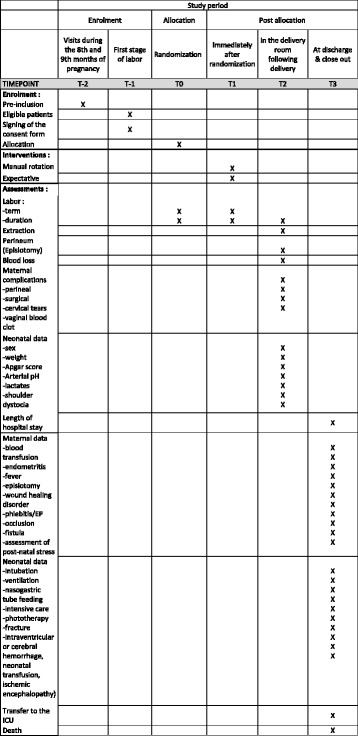
Flow chart

The participant selection flow chart will be provided (Fig. 2), as well as a descriptive analysis of study population characteristics.

For qualitative variables, results will be given in numbers and percentages, then compared using Pearson’s chi-squared test (or Fisher’s exact test). For quantitative variables, results will be reported as mean and standard deviation (or as a median with the 25th and 75th percentiles), then compared using Student’s *t* test (or the non-parametric Whitney-Mann *U* test). No intermediary analysis is foreseen in this study.

Analysis for the main objective: to achieve the primary objective, we will perform a test comparing the number of spontaneous vaginal deliveries in the two groups using Pearson’s chi-squared test (provided that the conditions for using this test are satisfactory in terms of numbers). In the event that this test cannot be performed, we will use Fisher’s exact test.

Analysis for the secondary objectives: to achieve the secondary objectives, i.e., assessing the impact of manual rotation on posterior positions at full dilation, we will use classical descriptive statistical tools, where the main parameters (mainly mean and percentage) will be accompanied by a 95% confidence interval. These observations will be reported using the most suitable type of diagram. Tests comparing means or numbers (percentages) may be performed, provided that their statistical power is satisfactory (1 − *β* > 80%). If it is not satisfactory, an observational approach will be used. The significance threshold will be set at 0.05 and all tests will be bilateral.

Analysis will be performed with the intention to treat: all participants whose endpoints will be available will be taken into account in the analysis according to their randomization group. No imputation method will be used in the event of missing data.

## Monitoring

### Data monitoring

A clinical research assistant (CRA) appointed by the sponsor will ensure that the study is conducted correctly and that data is appropriately collected, documented, recorded, and reported, in accordance with Good Clinical Practices and the legal and regulatory provisions in force.

### Harms

All serious adverse events (SEAs) that occur from the time of the patient’s inclusion to the time of discharge from the maternity ward will be declared to the sponsor, regardless of whether the SAEs relate to the patient included or to her newborn. Any SAEs that occur beyond that period will be declared to the sponsor only if the investigator attributes them to the experimental approach.

## Discussion

Given that the efficacy of manual rotation has not been proven with a high level of evidence, the practice of this technique is not systematically recommended by scholarly societies and is, therefore, rarely performed by obstetric gynecologists.

If our hypothesis regarding the superiority of manual rotation is confirmed, our study will help change delivery practices in cases of posterior fetal position. An increase in the rates of vaginal delivery will help decrease the short- and long-term rates of morbidity and mortality following cesarean section. Manual rotation is a simple and effective method with a success rate of almost 90%. Several preliminary studies have shown that manual rotation is associated with reduced rates for fetal extraction and maternal complications: Shaffer has shown that the cesarean section rate is lower in patients for whom a manual rotation is performed successfully (2%) with a 9% rate of cesarean sections when manual rotation is performed versus 41% when it is not performed. Le Ray has shown that manual rotation significantly reduces vaginal delivery rates via fetal extraction (23.2% vs 38.7%, p > 0.01). However, manual rotation is not systematically performed due to the absence of proof of its efficacy in retrospective studies and quasiexperimental before/after studies.

### Auditing

All SAEs that occur from the patient’s inclusion until discharge from the maternity ward will be declared in accordance with the procedures in force and an annual safety report will be carried out. However, given the low number of risks related to manual rotation reported in the academic literature for both mother and child, it was deemed unnecessary to set up an independent monitoring committee.

There will be no external audit.

### Trial status

At the time of manuscript submission, 87 patients have been included (22 November 2018).

## Additional file

Additional file 1: SPIRIT Checklist. (PDF 159 kb)

## Abbreviations

CRA: Clinical research assistant
CTG: Cardiotocogram
ICU: Intensive care unit
INSERM: Institut National de la Santé et de la Recherche Médicale
MRI: Magnetic resonance image
SEAs: Serious adverse events
SOGC: Society of Obstetricians and Gynecologists of Canada
WG: Weeks’ gestation
